# Expression of mouse CD47 on human cancer cells profoundly increases tumor metastasis in murine models

**DOI:** 10.1186/s12885-015-1980-8

**Published:** 2015-12-16

**Authors:** Armando Rivera, Xinping Fu, Lihua Tao, Xiaoliu Zhang

**Affiliations:** Department of Biology and Biochemistry and Center for Nuclear Receptors and Cell Signaling, University of Houston, Texas, USA

**Keywords:** Metastasis, Prostate cancer, PC-3 cells, Macrophage, CD47-SIRPα

## Abstract

**Background:**

Many commonly used xenograft tumor models do not spontaneously metastasize to distant organs following subcutaneous or orthotopic implantation, limiting their usefulness in preclinical studies. It is generally believed that natural killer cells are the key component of the innate immune system in determining tumor metastatic potential in xenograft models. However, recent studies suggest that macrophages may play an important role, as resident macrophages can eliminate the invading tumor cells if they do not express adequate levels of the CD47 molecule.

**Methods:**

We investigated the effect of overexpressing murine CD47 (mCD47) in PC-3 cells, a commonly used human prostate cancer line, on the metastatic potential in three mouse strains with different genetic background and varying degrees of immunodeficiency. We implanted the tumor cells either subcutaneously or orthotopically and then examined their local and distant metastases.

**Results:**

Our results show that mCD47-expressing PC-3 cells subcutaneously implanted in NSG and CB17. Scid mice metastasized to the sentinel lymph node, lung and liver significantly more efficiently than the control cells. When implanted orthotopically to NOD. Scid mice, these cells spontaneously metastasized to lung and liver.

**Conclusions:**

Our data demonstrate that mCD47 can facilitate human tumor cell metastasis in murine models, and that these mCD47-expressing tumor cells may be useful for in vivo studies where spontaneous metastases are desirable.

## Background

Xenografted tumor models are frequently used in cancer research, particularly for the purpose of evaluating experimental therapeutics. However, many of the commonly used xenograft tumor models do not spontaneously metastasize to other organ tissues after subcutaneous or orthotopic implantation [1]. This has diminished their usefulness in preclinical studies. Take prostate cancer as an example, many human prostate cancer cell lines have been established from patients [2]. Although most of them were primarily obtained from metastatic deposits, they usually exhibit a deficient metastatic behavior when implanted in immunodeficient mice [2]. Attempts to improve the metastatic potential of the commonly used prostate cancer cell lines seem to have only achieved a moderate success. For example, a few sublines with increased metastatic potential have been isolated from the PC-3 M and LNCaP human prostate cancer cells through multiple rounds of implanting these tumor cells intraprostatically followed by harvesting tumor tissues from the draining lymph nodes [3]. Although some of these sublines (e.g. PC-3 M-LN4) produced higher incidence of lung and bone metastasis after intravenous or intracardiac inoculation, they did not show significant enhancement over the parental line on spontaneous metastasis after orthotopic implantation [3].

Spontaneous cancer metastasis is a multi-step process, in which the tumor cells in their primary site initially become detached from the local tumor tissue. The detached tumor cells subsequently enter into either blood or lymphatic circulation, which is followed by extravasation into the distant organ tissue. After arriving in the parenchyma of the secondary organ, the tumor cells need to adapt to the new environment to reinitiate their growth for the eventual formation of micrometastases [4]. Studies have suggested that interaction of the arrived tumor cells, the “seeds”, with the local tissues of the metastatic site, the “soils”, is a complex process [5]. For example, some studies have suggested that primary tumors may release systemic signals to allow establishment of specific niches at the metastatic site to accommodate the metastatic propensity of the extravasated tumor cells [6, 7]. Other studies have shown that the arrived tumor cells can actively adapt to the new tissue microenvironment at the metastatic site by altering their gene expression such as activation of the Src tyrosine kinase signaling [8]. More recent studies have suggested that local macrophages may be an important “soil” component in dictating the ability of the arrived tumor cells to form metastatic tumor, as the resident macrophages have the capability to eliminate the invading tumor cells if they do not express an adequate level of the “don’t eat me” signal – the CD47 molecule [9–11].

In this study, we investigated the effect of overexpressing murine CD47 (mCD47) in the commonly used PC-3 human prostate cancer cell line on its metastatic potential in murine models. CD47 is a membrane protein and the ligand for SIRPα, a receptor expressed on phagocytic cells such as macrophages [11]. The binding of CD47 to SIRPα results in the inhibition of macrophage-mediated scavenging and phagocytosis, which renders CD47 the moniker of “don’t eat me” signal [12]. We hypothesized that overexpression of mCD47 would allow human cancer cells to escape detection and elimination by macrophages and possibly some other phagocytes, which are largely intact in immune deficient mice used for xenografted tumor models. This should allow the xenografted tumor cells to grow better with more efficient metastasis in murine models. We tested our hypothesis in three mouse strains with a varying degree of immunodeficiency and different genetic background. Our results show that mCD47-expressing PC-3 cells implanted on the flank of NSG mice were able to metastasize to the sentinel lymph nodes, lung and liver in a significantly greater extent than the control cell lines. When implanted orthotopically to the NOD.Scid mice, the mCD47-expressing PC-3 cells spontaneously metastasized to lungs and livers more efficiently and formed larger tumors when compared with the control PC-3 and PC-3 M-LN4 cells. Similar results were obtained with the cells implanted in CB17.Scid mice. Our data thus demonstrate that overexpression of mCD47 can enhance human tumor cell metastasis in murine models, and that these mCD47-expressing tumor cells can be useful for in vivo studies where spontaneously metastases are desirable.

## Methods

### Cancer cell lines and cultures

The human prostate cancer cell line PC-3 (derived from a bone metastasis), the human colon cancer cell line HCT116 and the immortalized murine macrophage cell line RAW 264.7 were obtained from the American Type Culture Collection (Manassas, VA). The PC-3 M-LN4, a PC-3 variant, was kindly provided by Dr. Isaiah J. Fidler (M. D. Anderson Cancer Center, Houston, TX). PC-3 cells were grown in complete RPMI 1640 medium supplemented with 20 mM HEPES, 2 mM L-glutamine, 10,000 U.I./mL penicillin-streptomycin, 1 mM sodium Pyruvate, 1 % MEM non-essential amino acids (Life Technologies, Grand Island, NY), and 10 % FBS (Fetal Bovine Serum, Gemini Bio-Products, West Sacramento, CA). HCT116 cells were grown in 10 % DMEM and 1 % penicillin-streptomycin. RAW 264.7 cell were cultured in DMEM containing 10 % heat-inactivated FBS and 1 % penicillin-streptomycin. Cells were cultured at 37 °C in a 5 % CO_2_ water saturated atmosphere.

### Generation of CD47-containing vectors for establishing stable cell lines

**Fig. 1:**
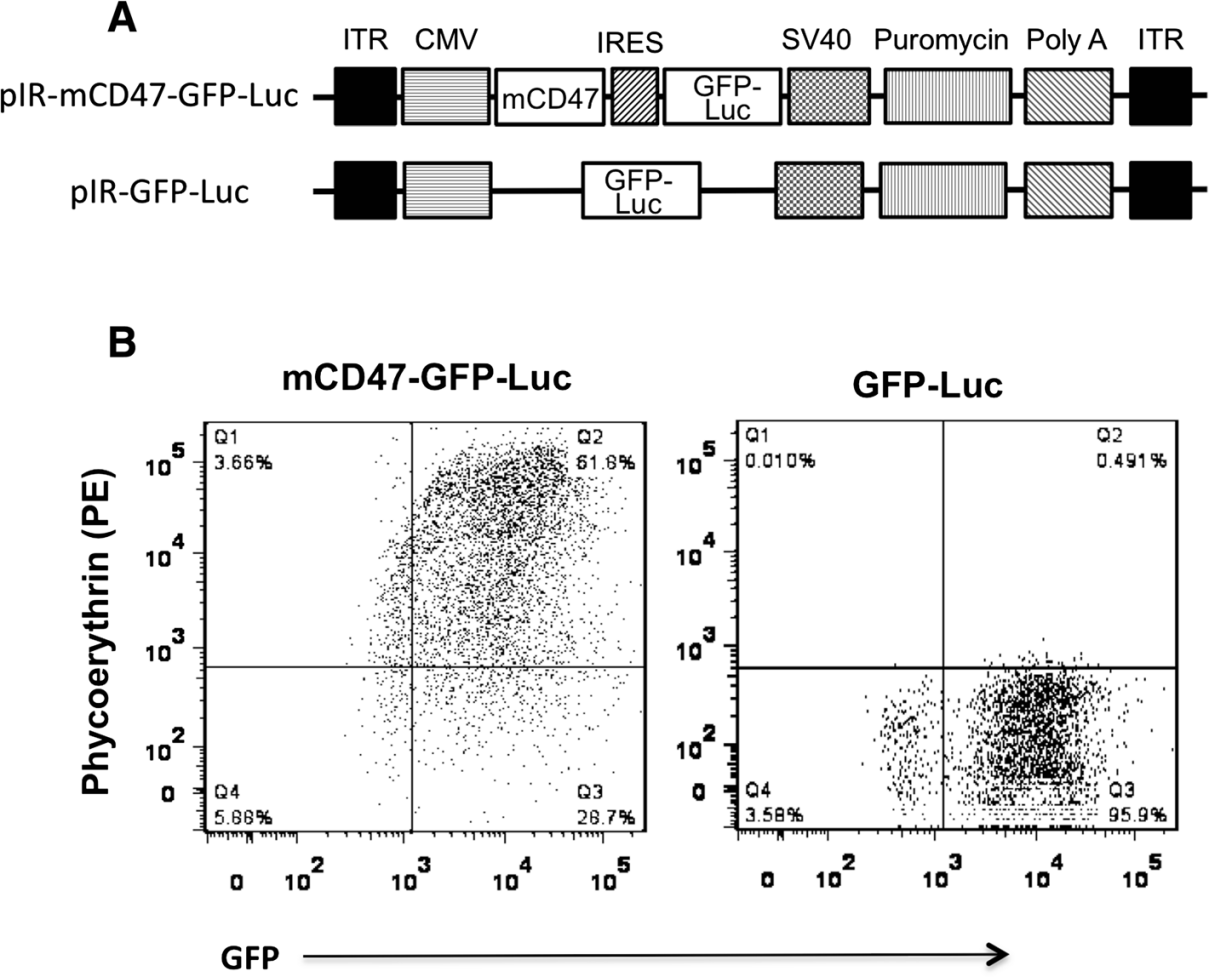
Construction and expression of mCD47. **a** Schematic illustration of mCD47-GFP-Luc and GFP-Luc in a transposon vector construct. The coding sequences for murine CD47 (mCD47) or GFP-luciferase fusion gene (GFP-Luc) are shown. The locations of the promoter, the polyA signal, and the internal ribosome entry site (IRES) are indicated. **b** Transfection efficiency of mCD47. PC-3 cells were transfected with a Piggyback transposon with mCD47- GFP-Luc or GFP-Luc vectors, or mock transfected. Following puromycin selection, cells were stained with an isotype or a PE-conjugated anti-mCD47 antibody, and then analyzed by flow cytometry for detection of PE and GFP

The murine CD47 coding sequence (GenBank: Z25524.1) was synthesized by Integrated DNA Technologies (Coralville, IA), and cloned into the mammalian expression vector pIR-PURO. A fusion gene of GFP and luciferase, which was generated in our own lab, was also cloned into the same pIR-PURO plasmid (Fig. 1). The details of plasmid pCMVpiggyBAC have been described elsewhere [13]. It was transfected together with pIR-GFP-Luc or pIR-PURO-mCD47 (Fig. 1a) into either PC-3 or HCT116 cells, using Lipofectamine 2000 and Opti-MEM (Life Technologies, Grand Island, NY). Puromycin was added to transfected cells 48 h later at a concentration of 1 μg/mL, which was gradually increased up to 3 μg/mL for two weeks, as previously described for resistant-cell selection (Rivera et al., 2011).

### Flow cytometric analysis and cell sorting

The puromycin-resistant, GFP-, and CD47-expressing PC-3 cells were labeled for FACS analysis (GFP and CD47 double positive cells) with either a rat PE Rat IgG2a, κ isotype control antibody (cat # 400507, Biolegend CA) or a rat PE anti mouse CD47 antibody (Cat # 127507, Biolegend). For live sorting, a concentration of ≤1.0 μg per million cells in 100 μl volume was used. Cells were incubated for 30 min at 4 °C in the dark in a PBS solution containing 2 % FBS plus either the isotype or the mCD47 antibody. Cells then were washed twice with the same solution. FACS analysis was performed on a Beckton Dickinson FACSAria (BD Biosciences, San Jose, CA). Gates were established with unstained cells operating at low pressure (20 psi) using a 100 μm nozzle. Cell clusters and doublets were electronically gated out. Cells were routinely double-sorted using GFP and PE, and post-sort analysis typically indicated purities of > 90 % with minimal cell death (<10 %). Total viable cells were used for the following experiments.

### Phagocytosis assay

For the *in vitro* phagocytosis assay, Raw 264.7 cells were first activated with 50U/ml of murine IFNγ and 10 ng/ml of LPS for 24 h. The cells were then plated into a 96-well at 1 × 10^5^ per well along with 2 × 10^4^ cancer cells. The next day, phagocytosis was verified by the luciferase assay using the Bright-Glo™ Luciferase Assay System (cat # E2610, Promega, Madison, WI) according to the manufacture’s instruction. Briefly, wells were rinsed with PBS; after that, 200 μl of a 1:1 mix of PBS plus Bright-Glo reagent were poured into each well, mixed with the cells and then luciferase activity was measured in a Victor X4 Multilabel Plate Reade spectrophotometer (Perkin Elmer, Waltham MA).

### Animal studies

All animal husbandry and experimental procedures conducted in this study were approved by the University of Houston Institutional Animal Care and Use Committee (IACUC). Six-week-old male NOD.Cg-Prkdc^scid^Il2rg^tm1Wjl^/SzJ mice or NSG mice, NOD.CB17-*Prkdc*^*scid*^*/*J or NOD.Scid mice (The Jackson Laboratory, Bar Harbor, ME), and C.B-*Igh-1*^*b*^/IcrTac-*Prkdc*^*scid*^ or CB17.Scid (Taconic, Germantown, NY) were used in this study. The stable human prostate cancer cell lines PC-3 expressing either mCD47-GFP-Luc (PC3-mCD47) or GFP-Luc (PC3-GFP-Luc) were implanted subcutaneously into the mouse right flank in a concentration of 2 × 10^6^ cells. PC3-mCD47, PC3-GFP-Luc or PC-3 M-LN4 were implanted orthotopically in a concentration of 2 × 10^4^ cells.

Tumors implanted subcutaneously were let grow for up to 3 or 4 weeks or until they reached approximately 1500 mm^3^ and then excised. Tumor growth was monitored every 3 days by measuring two perpendicular tumor diameters with a caliper, and their volume was calculated by the formula ½ (Length × Width^2^). For the orthotopic model, each mouse received intraprostatic injections of PC3-mCD47, PC3-GFP-Luc or PC-3 M-LN4. Bioluminescent imaging was done weekly for a month to quantitate the luciferase signal from PC3-mCD47 and PC3-GFP-Luc cells as described in more detail in the following section. Mice were sacrificed after that, lungs and livers were harvested and metastatic lesions on these organs were counted after H&E staining.

For *in vivo* imaging, mice were fed on alfalfa-free rodent food (Teklad Global irradiated Soy Protein-Free Extruded Rodent Diet Cat # 2920X, Harlan Laboratories, Madison WI) two weeks prior and during imaging. After tumor excision, weekly observation of the luciferase activity was performed sequentially for a month using an IVIS Spectrum Pre-clinical in vivo Imaging System (Perkin Elmer, Waltham MA). Mice were injected intraperitoneally with 150 mg/kg D-luciferin (cat # LUCK-1G, Gold Biotechnology, St. Louis, MO) dissolved in water. Bioluminescence images were taken 5–10 min after the luciferin injection. A negative control mouse injected with luciferin was placed next to treated animals during each image acquisition to provide a constant reference for the background. Images were analyzed using Living Image version 4.2 software (Perkin Elmer) and represented as total flux measurements in photons/second.

For histological staining, organ tissues including lungs and livers were collected and fixed in 10 % formalin. Serial 5-μm cross-sections of pulmonary and hepatic metastases from mice implanted with either PC3-mCD47 or PC3-GFP-Luc cells were prepared and H&E stained for examination with light microscopy. Five fields of each single representative section were examined for each organ from each of the five mice in each group using an Olympus BX51 microscope, a camera Olympus DP73, and its associated software, Olympus cellSens® 1.9 (Olympus Imaging America Inc., Center Valley, PA).

### Statistical analysis

All quantitative data are reported as mean ± SD. Statistical analysis was made for multiple comparisons using analysis of variance and Student’s t-test. *p* value <0.05 was considered to be statistically significant.

## Results

### Establishment and characterization of mCD47-expressing PC-3 cells

The coding sequence for murine CD47 was synthesized according to GenBank (Z25524.1), and cloned into the transposon vector pIR-PURO [14]. To facilitate *in vitro* and *in vivo* characterization, a fusion gene of GFP luciferase (GFP-Luc) was included in all the transposon vectors, with the control vector containing GFP-Luc only. The detailed composition of these transposon vectors is depicted in Fig. 1a. To ensure that the established cells overexpress the transgenes, pIR-mCD47-GFP-Luc or pIR-GFP-Luc was co-transfected with pCMVpiggyBAC [13]. The piggyBac transposase expressed from pCMVpiggyBAC would allow the transgenes contained in the transposon donor plasmids to be efficiently integrated into the chromosomes of PC-3 cells at multiple copy numbers, ensuring a high level of transgene expression [13, 15]. The cells were initially selected by puromycin treatment followed by flow cytometry sorting as described [14]. Expression of mCD47 was confirmed by flow cytometry (Fig. 1b). The newly established cell lines are designated PC3-mCD47 and PC3-GFP-Luc (the control cell line), respectively.

**Fig. 2:**
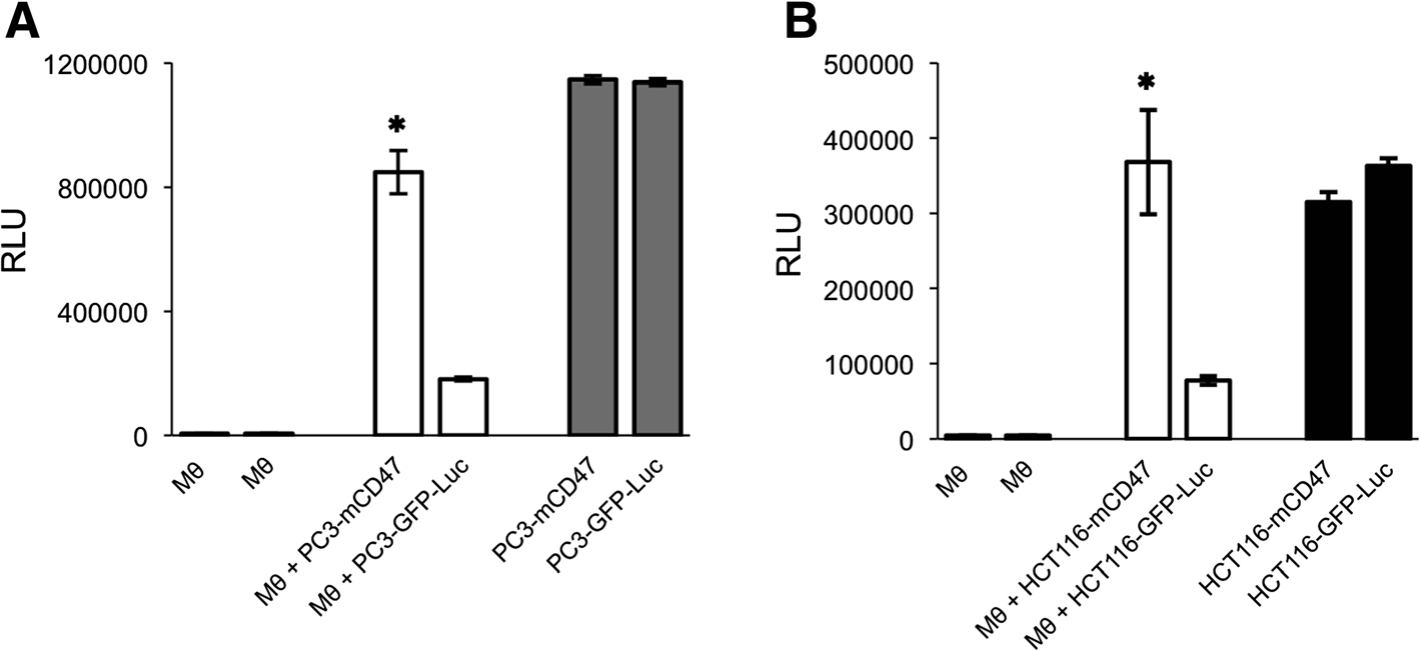
*In vitro* assay on phagocytic effect of activated macrophages against human tumor cells with or without mCD47 expression. Activated RAW 264.7 mouse macrophages were co-cultured with either PC3-mCD47 and PC3-GFP-Luc cell pair (**a**) or HCT116-mCD47 and HCT116-GFP-Luc cell pair (**b**). Tumor cell viability was determined by measuring intracellular luciferase activity and was presented as relative light unit (RLU). Cancer cells without macrophages co-culturing (*the last column of each figure*) were used as a positive control and macrophages without tumor cells were used as the negative control for the assay. Bars represent means plus standard deviations for two experiments, each with duplicate samples. ✱*p* < 0.05 as compared to the respective GFP-Luc control cells

We further characterized the PC3-mCD47 cells by comparing them with PC3-GFP-Luc control cells for their sensitivity to the phagocytic effect of activated macrophages. To determine the generality of the impact from mCD47 overexpression, we included in this experiment another tumor cell that was similarly transduced with mCD47 but is from different tissue origin. HCT116 is a human colon cancer cell line and it was transduced with the same transposon constructs to establish HCT116-mCD47 and HCT116-GFP-Luc. The tumor cells were mixed with activated macrophages and, as all the tumor cells express luciferase, their viability was conveniently determined by measuring intracellular luciferase activity after the cells were extensively washed. The results show that the viability of both PC3-mCD47 and HCT116-mCD47 was significantly higher than that of their respective control cells (Fig. 2). These results show that overexpression of mCD47 in human cells can negatively impact the phagocytic effect of murine macrophages when they encounter each other.

### Overexpression of mCD47 only marginally enhances local tumor growth but greatly increase tumor metastasis to the sentinel lymph node and distant organs in NSG mice

**Fig. 3:**
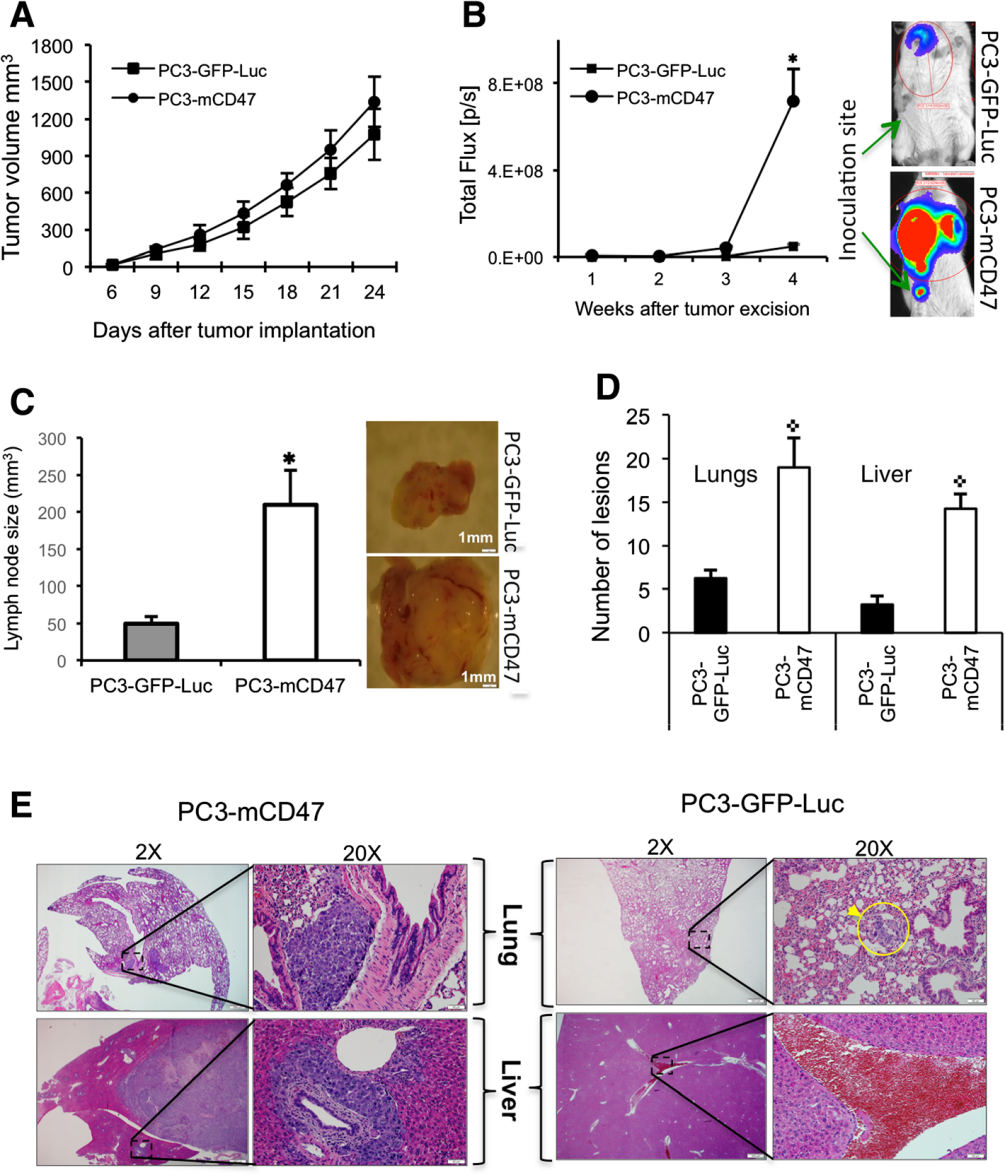
Local tumor growth and metastasis to the draining lymph node, lung and liver in NSG mice. 2 × 10^6^ PC3-mCD47 and PC3-GFP-Luc cells were injected to the right flank of NSG mice and tumor growth was monitored for 24 days **a**. Then primary tumor was surgically removed and animals were optically imaged weekly with IVIS for luciferase. The signal detected within the region of interest (indicated with a red circle) covers the draining lymph node and other distant organs such as lung and liver **b**. Shown on the right-hand side of B are typical images from each animal group, in which the original tumor inoculation sites were indicated by arrows. The draining lymph node was collected and measured four weeks later after termination of the experiment **c**. Shown on the right-hand side of C are representative gross appearances of the explanted draining lymph nodes. **d** By the end of the experiment, mice were euthanized and lung and liver were collected for preparation of tissue sections, which were then H&E stained for histological examination and quantitation of metastatic lesions. The number of metastatic lesions was obtained from counting five different fields of organ sections from each animal. **e** shows representative micrographs from the H&E stained lung and liver tissue. Low magnification showing the entire organ section is on the left of each panel, and high magnification of the defined square area showing metastatic lesions is on the right of each panel. The metastatic lesions are indicated with arrowheads. Error bars represent the mean ± SD (*n* = 5 per group), ✱*p* < 0.05 and ✜ *p* < 0.01 as compared to PC3-GFP-Luc

To compare tumor growth properties, both PC3-GFP-Luc and PC3-mCD47 cells were implanted subcutaneously to the right flank of NSG mice. Local tumor growth was measured every 3 days. The size of PC3-mCD47 tumor was only marginally bigger than that of PC3-GFP-Luc in NSG mice (Fig. 3a). The local tumors were then surgically removed to allow the monitoring of tumor cell metastasis to the sentinel lymph node and distant organs by IVIS optical imaging. The image signal (radiance in p/s unit) became detectable at week 3 in mice implanted with PC3-mCD47 cells, mainly in the body area that covers the draining lymph node and some distant organs including lung and liver (Fig. 3b). The radiance then got increasingly stronger, and by week 4, it reached to an extremely high level while the radiance in mice planted with PC3-GFP-Luc was only barely detectable. The experiment was terminated at this point, and the draining lymph node, lung and liver were collected. The sentinel lymph nodes in mice implanted with PC3-mCD47 cells were vastly enlarged. Their average size is 4 times larger than that in mice implanted with PC3-GFP-Luc cells (Fig. 3c). Microscopic examination of the sections prepared from the collected lung and liver revealed significantly more and larger metastatic lesions in mice implanted with PC3-mCD47 cells than in the PC3-GFP-Luc group (Fig. 3d and e). These data demonstrate that, although overexpression of mCD47 in PC-3 cells only marginally enhances the establishment of local tumors, it significantly potentates the spontaneous metastasis of tumor cells to the sentinel lymph node and distant organs including lung and liver.

### mCD47 also increases metastatic potential of PC-3 cells in NOD.Scid mice following orthotopic implantation

**Fig. 4:**
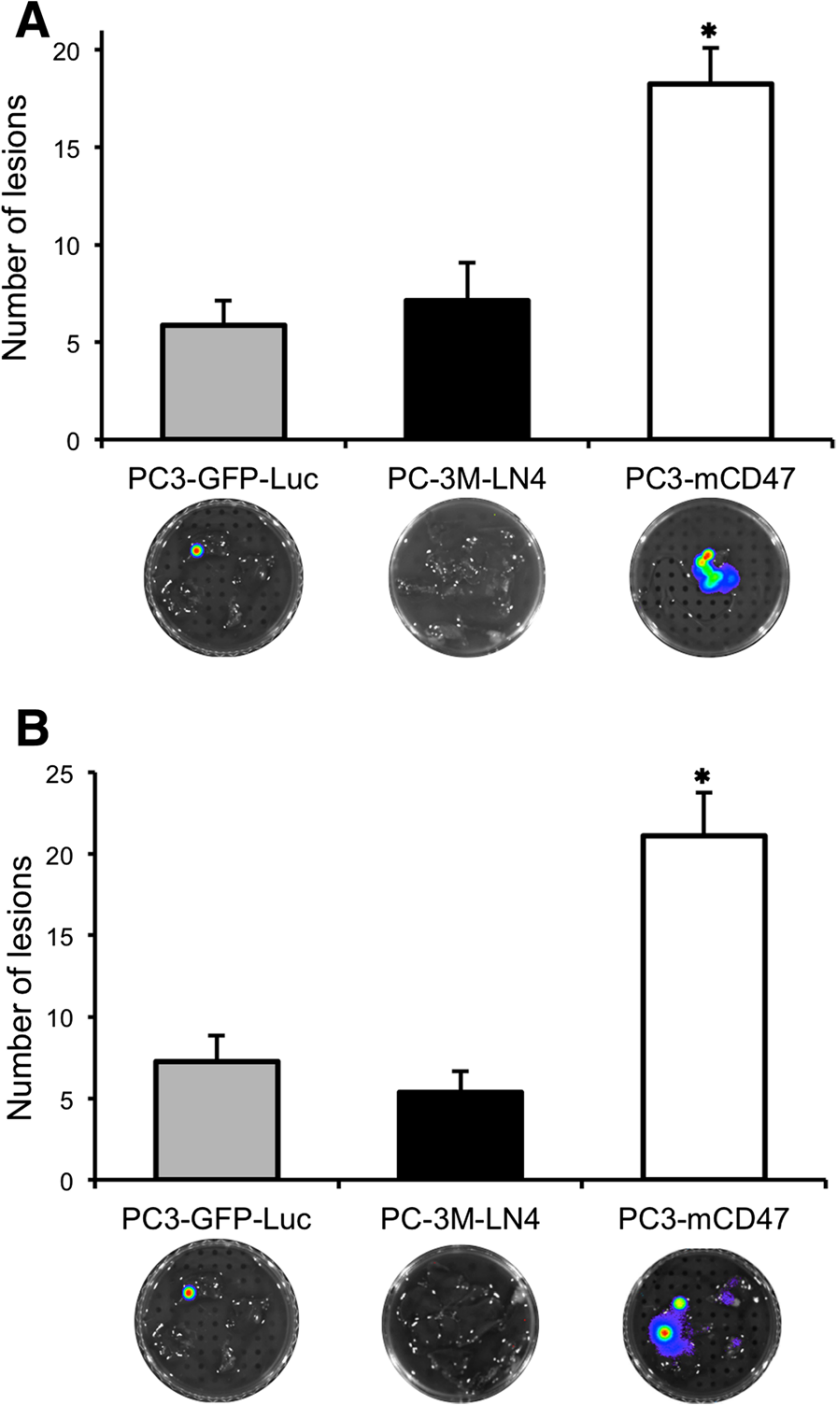
Distant metastasis after orthotopic implantation of tumor cells in NOD.Scid mice. PC3-mCD47, PC3-GFP-Luc and PC-3 M-LN4 cells were orthotopically implanted to the prostates of male NOD.Scid mice and orthotopic tumor formation in the former two groups was monitored and confirmed by weekly bioluminescent imaging (data not shown here). Five weeks after tumor cell implantation, mice were euthanized. Lungs (**a**) and livers (**b**) were collected and were initially imaged for luciferase signal intensity before they were sectioned and H&E stained for microscopic detection of metastatic lesions as described in Fig. 3d. The luciferase images were presented underneath the corresponding column in each figure. ✱*p* < 0.05 as compared to the other two groups

Due to the presence of additional IL2 receptor gamma chain deletion, the immunodeficiency in NSG mouse was significantly more severe than that of NOD.Scid mouse [16]. Consequently, some human tumor cells show an improved metastatic potential in NSG mouse [17]. Thus, it is desirable to determine if PC3-mCD47 cells also have an increased metastatic property in the less immunodeficient NOD.Scid mice. Additionally, it is also important to determine if the enhanced metastatic property of PC3-mCD47 cells could be recapitulated following orthotopic route of implantation. As such, we implanted both PC3-GFP-Luc and PC3-mCD47 cells intraprostatically into the NOD.Scid mice. We also added an additional group of animals in which the more metastatic PC-3-LN4 cells were implanted by the same orthotopic route so that the metastatic outcome could be compared among the different groups. Bioluminescent imaging was done weekly on mice implanted with PC3-mCD47 and PC3-GFPLuc cells to monitor and confirm tumor growth at the orthotopic site (data not shown). Mice were euthanized five weeks after the orthotopic tumor cell implantation and lungs and liver were harvested. Following H&E staining, the metastatic lesions were enumerated as in Fig. 3d. The results show that animals orthotopically implanted with PC3-mCD47 cells had 2.5- and 3-fold more pulmonary lesions than the mice implanted with PC3-GFP-Luc and PC-3 M-LN4 cells, respectively (Fig. 4a). The same metastatic trend was detected in the liver, with mice in the PC3-mCD47 group having 3- and 4-fold more hepatic lesions than mice in PC3-GFP-Luc and PC-3 M-LN4 group, respectively (Fig. 4b). Interestingly, our data also showed that PC-3 M-LN4 cells were not significantly more metastatic than PC3-GFP-Luc cells after orthotopic implantation, which is in line with the observation made by other groups [3]. Together, these data suggest that PC3-mCD47 cells have a superior ability to spontaneously metastasize to distant organ tissues than the control PC3-GFP-Luc cells as well as the selected metastatic PC-3 M-LN4 cells following orthotopic implantation.

### mCD47 promotes spontaneous metastasis in CB17.Scid mouse in which the parental cells fail to metastasize

**Fig. 5:**
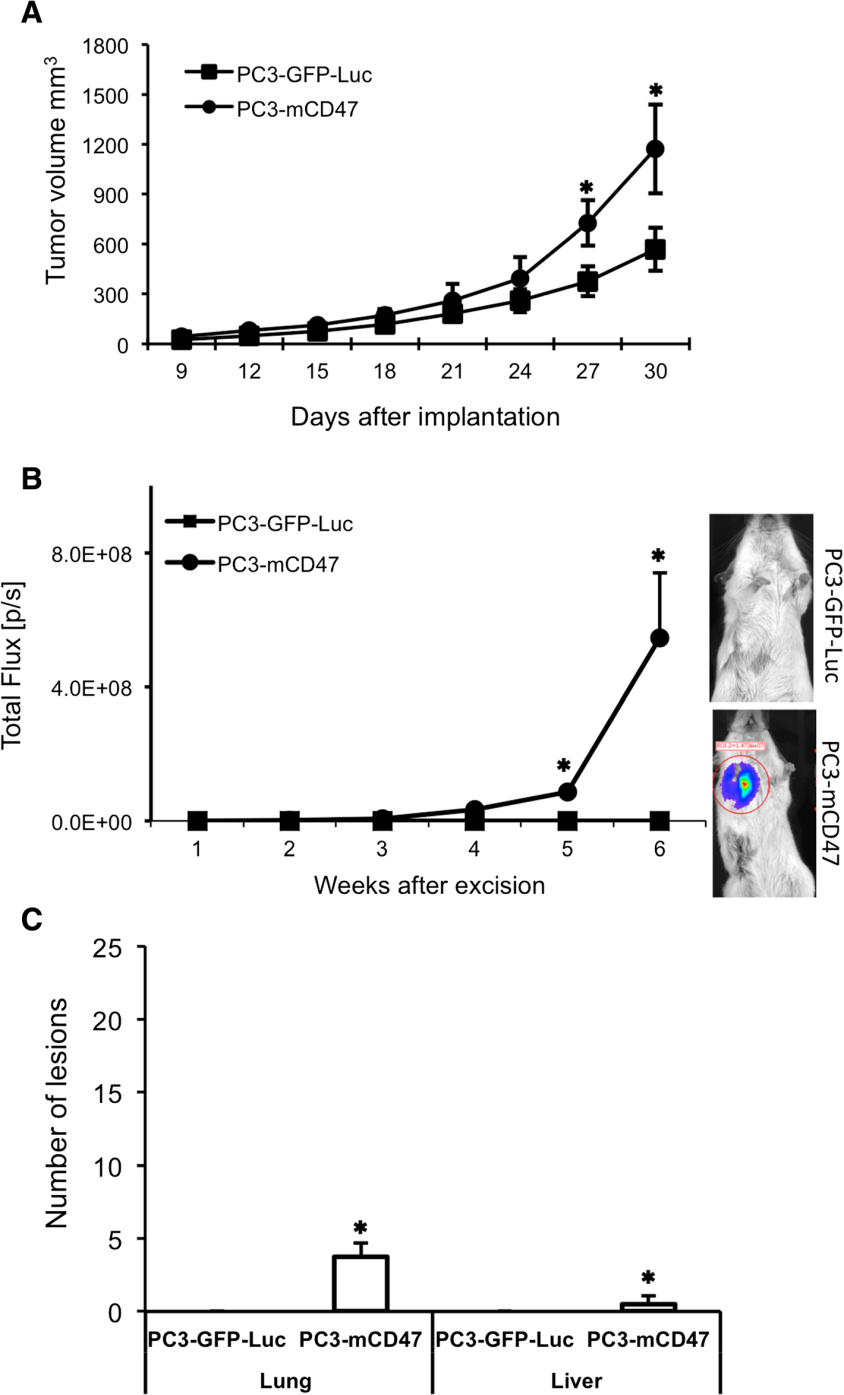
Comparison of local tumor growth and distant metastasis in CB17.Scid mice. PC3-mCD47 and PC3-GFP-Luc cells were implanted subcutaneously to the right flank of CB17.Scid mice. Tumor growth was measured periodically for four weeks (**a**) before the tumors were excised. Then the animals were imaged weekly for bioluminescent signal (**b**) for six weeks before they were euthanized for collection of lungs and livers. Shown on the right-hand side of B are typical images from each animal group. Metastatic lesions in the lung and liver were determined by microscopic examination of H&E stained tissue sections (**c**). ✱*p* < 0.05 as compared to PC3-GFP-Luc

Although mCD47 was found to enhance metastases of PC-3 cells to the distant organs in both NSG and NOD.Scid mice after subcutaneous or orthotopic implantation, the control PC3-GFP-Luc cells showed metastases to these organs as well albeit at a significantly lower efficiency (Figs. 3 and 4). It has been reported that CB17.Scid mouse was significantly less permissive than NOD.Scid in supporting the engraftment of human hematopoietic stem cells [18, 19]. One of the reasons for the difference on this permissiveness is that, due to the SIRPα polymorphism among different mouse strains, SIRPα in Balb/c genetic background (from which CB17.Scid is derived) has a much lower binding affinity than its counterpart in C57BL/6 (from which NOD.Scid is derived) to human CD47 [19]. As such, we also implanted both PC3-mCD47 and PC3-GFP-Luc cells subcutaneously to CB17.Scid mice to compare the local tumor growth as well as migration to the draining lymph nodes and metastasis to the distant organs. The results show that, unlike in NSG and NOD-Scid mice, the control PC3-GFP-Luc cells completely failed to migrate to the draining lymph node and to metastasize to either lung or liver (Fig. 5b and c). Expression of mCD47 moderately enhanced local tumor growth (Fig. 5a) but significantly potentiated the tumor cell migration to the draining lymph node (Fig. 5b) and distant metastasis to the lung (Fig. 5c). Liver metastasis was detected in the mice from PC3-mCD47 group, but at a low frequency. These data demonstrate that mCD47 expression could enable PC-3 tumor cells to initiate spontaneous local and distant metastasis in a mouse strain in which otherwise the tumor cells would not be able to metastasize.

## Discussion

Lack of reliable and efficient metastatic xenograft cancer models is hampering the progress of many preclinical studies, particularly for the purpose of drug evaluation. In this study, we have examined the effect of overexpressing murine CD47 on the metastatic potential of a widely used human pancreatic cell line. This study was partly prompted by the recent demonstration that CD47 is a key “don’t eat me” signal, as masking CD47 on tumor cells with monoclonal antibodies allow macrophages to efficiently clear the malignant cells [9]. As human CD47 does not cross react with murine SIRPα and macrophages in most immunodeficient mice remain largely intact [20], we reasoned that lack of engagement of CD47- SIRPα might be a contributing factor for the low metastatic potential of many xenografted tumors. Our data indeed showed that overexpression of mCD47 in the popular PC-3 human prostatic cancer cell line could increase the tumor cell metastasis to the distant organs such as the lung and liver after the tumor cells were implanted either subcutaneously or orthotopically. In fact, under direct comparison, PC3-mCD47 cells produced a significantly more spontaneous metastasis to the distant organs of lung and liver than the well-established metastatic subline PC-3 M-LN4 in NOD.Scid mice, after both cells were orthotopically implanted. Moreover, mCD47 expression enables PC-3 tumor cells to initiate spontaneous local and distant metastasis in a mouse strain (CB17.Scid) that otherwise is resistant to the metastasis of the tumor cell. As such, in addition to support the notion that proper engagement of CD47 and SIRPα is important for establishment of metastatic tumors, this study also suggests that PC3-mCD47 cells may represent a better metastatic model than PC-3 M-LN4 for preclinical investigations.

The conclusion drawn from this study is well supported by the literature. First, Willingham et al have evaluated CD47 expression on dissociated patient ovarian, breast, colon, bladder, glioblastoma, hepatocellular carcinoma, and prostate tumor cells. Their results show that CD47 is detected at an elevated level on nearly all these cancer cells [9]. The same study also demonstrates that *CD47* mRNA expression levels predict patient survival. More specifically, PC-3 cells have been found to overexpress CD47 [21]. Second, studies by Ide et al have shown that the interspecies incompatibility of CD47 contributes to the rejection of xenogeneic cells by macrophages [22]. Third, other studies have shown that there is an extensive crosstalk between colon cancer cells and macrophages and that CD47 promotes tumor cell migration [23]. Finally, studies in combination with clinical data have shown that overexpression of CD47 on circulating tumor cells correlates with dismal survival and increased metastasis in a small cohort of metastatic luminal breast cancer patients [24, 25]. Our study is the first direct demonstration that overexpression of murine CD47 in human cancer cells can potentiate the metastatic potential of the tumor cells in the murine model. Although we only tested this in a single cell line, we believe that this principle can be applied to other commonly used popular human tumor cell lines.

We conducted the experiments in three mouse strains with different genetic background and varying degrees of immunodeficiency. NSG mouse contains all the immunodeficiency features of NOD.Scid and CB17.Scid with additional deletion in IL-2 gamma chain. As a consequence, all the mouse strains share the immunodeficiency of lacking T and B cells, with the major difference being that NSG mouse also lacks functional NK cells [16]. Our data show that overexpression of murine CD47 potentiates the metastatic potential of PC-3 cells to a similar extent in all the three mouse strains, suggesting that macrophages probably play a more important role than NK cells in dictating the metastatic potential of PC-3 cells.

NSG and NOD.Scid mice share the same C57BL/6 genetic background. CB17.Scid, on the other hand, is derived from Balb/c and thus has a different genetic background as the other two. Studies have shown that murine SIRPα receptor from mouse strains with C57BL/6 genetic background can bind to human CD47 to a certain extent, while SIRPα from mice with Balb/c genetic background has very weak or no binding affinity to human CD47 [19]. This may have partly explained our observation that the control PC3-GFP-Luc cells showed a measurable metastasis in both NSG and NOD.Scid mice but completely failed to metastasize in CB17.Scid mice. This is despite the fact that CB17.Scid and NOD.Scid share a similar degree of immunodeficiency. The demonstration that mCD47 enables PC-3 tumor cells to initiate spontaneous local and distant metastasis in CB17.Scid mouse that otherwise is resistant to the metastasis of the tumor cell reinforces the notion that CD47-SIRPα mediated action of macrophage probably plays a more important role than other innate immune components in determining the metastatic potential of xenograft tumors.

## Conclusions

In conclusion, we provide evidence that proper engagement of CD47 with its receptor SIRPα is crucial for successful establishment of tumor metastases. Specifically, overexpression of murine CD47 can profoundly enhance the metastatic potential of human tumor cells in the xenografted tumor models. Due to the vastly improved metastatic potential, these established cell lines should be useful for preclinical studies in where a reliable and spontaneous metastatic tumor is desirable.

## Abbreviations

IRES: Internal ribosome entry site
mCD47: Murine CD47
SIRPα: Signal regulatory protein α, a receptor expressed on phagocytic cells such as macrophages

## References

[1] Khanna C, Hunter K (2005). Modeling metastasis in vivo. Carcinogenesis.

[2] Russell PJ, Kingsley EA (2003). Human prostate cancer cell lines. Methods Mol Med.

[3] Pettaway CA, Pathak S, Greene G, Ramirez E, Wilson MR, Killion JJ (1996). Selection of highly metastatic variants of different human prostatic carcinomas using orthotopic implantation in nude mice. Clin Cancer Res.

[4] Valastyan S, Weinberg RA (2011). Tumor metastasis: molecular insights and evolving paradigms. Cell.

[5] Langley RR, Fidler IJ (2011). The seed and soil hypothesis revisited--the role of tumor-stroma interactions in metastasis to different organs. Int J Cancer.

[6] Kaplan RN, Riba RD, Zacharoulis S, Bramley AH, Vincent L, Costa C (2005). VEGFR1-positive haematopoietic bone marrow progenitors initiate the pre-metastatic niche. Nature.

[7] Psaila B, Lyden D (2009). The metastatic niche: adapting the foreign soil. Nat Rev Cancer.

[8] Zhang XH, Wang Q, Gerald W, Hudis CA, Norton L, Smid M (2009). Latent bone metastasis in breast cancer tied to Src-dependent survival signals. Cancer Cell.

[9] Willingham SB, Volkmer JP, Gentles AJ, Sahoo D, Dalerba P, Mitra SS (2012). The CD47-signal regulatory protein alpha (SIRPa) interaction is a therapeutic target for human solid tumors. Proc Natl Acad Sci U S A.

[10] Weiskopf K, Ring AM, Ho CC, Volkmer JP, Levin AM, Volkmer AK (2013). Engineered SIRPalpha variants as immunotherapeutic adjuvants to anticancer antibodies. Science.

[11] Barclay AN, Van den Berg TK (2014). The interaction between signal regulatory protein alpha (SIRPalpha) and CD47: structure, function, and therapeutic target. Annu Rev Immunol.

[12] Jaiswal S, Jamieson CH, Pang WW, Park CY, Chao MP, Majeti R (2009). CD47 is upregulated on circulating hematopoietic stem cells and leukemia cells to avoid phagocytosis. Cell.

[13] Wilson MH, Coates CJ, George AL (2007). PiggyBac transposon-mediated gene transfer in human cells. Mol Ther.

[14] Rivera A, Fu X, Tao L, Zhang X (2011). Modification of a popular syngeneicmurinemammary tumor model for immunotherapy studies. ISRN Immunology.

[15] Chen X, Cui J, Yan Z, Zhang H, Chen X, Wang N (2015). Sustained high level transgene expression in mammalian cells mediated by the optimized transposon system. Genes Dis.

[16] Shultz LD, Lyons BL, Burzenski LM, Gott B, Chen X, Chaleff S (2005). Human lymphoid and myeloid cell development in NOD/LtSz-scid IL2R gamma null mice engrafted with mobilized human hemopoietic stem cells. J Immunol.

[17] Sartelet H, Durrieu L, Fontaine F, Nyalendo C, Haddad E (2012). Description of a new xenograft model of metastatic neuroblastoma using NOD/SCID/Il2rg null (NSG) mice. In Vivo.

[18] Larochelle A, Vormoor J, Hanenberg H, Wang JC, Bhatia M, Lapidot T (1996). Identification of primitive human hematopoietic cells capable of repopulating NOD/SCID mouse bone marrow: implications for gene therapy. Nat Med.

[19] Takenaka K, Prasolava TK, Wang JC, Mortin-Toth SM, Khalouei S, Gan OI (2007). Polymorphism in Sirpa modulates engraftment of human hematopoietic stem cells. Nat Immunol.

[20] Subramanian S, Parthasarathy R, Sen S, Boder ET, Discher DE (2006). Species- and cell type-specific interactions between CD47 and human SIRPalpha. Blood.

[21] Liu AY (2000). Differential expression of cell surface molecules in prostate cancer cells. Cancer Res.

[22] Ide K, Wang H, Tahara H, Liu J, Wang X, Asahara T (2007). Role for CD47-SIRPalpha signaling in xenograft rejection by macrophages. Proc Natl Acad Sci U S A.

[23] Zhang Y, Sime W, Juhas M, Sjolander A (2013). Crosstalk between colon cancer cells and macrophages via inflammatory mediators and CD47 promotes tumour cell migration. Eur J Cancer.

[24] Baccelli I, Schneeweiss A, Riethdorf S, Stenzinger A, Schillert A, Vogel V (2013). Identification of a population of blood circulating tumor cells from breast cancer patients that initiates metastasis in a xenograft assay. Nat Biotechnol.

[25] Baccelli I, Stenzinger A, Vogel V, Pfitzner BM, Klein C, Wallwiener M (2014). Co-expression of MET and CD47 is a novel prognosticator for survival of luminal breast cancer patients. Oncotarget.

